# Effect of Sandblasting on Static and Fatigue Strength of Flash Butt Welded 75Cr4 Bandsaw Blades

**DOI:** 10.3390/ma14226831

**Published:** 2021-11-12

**Authors:** Andrzej Kubit, Łukasz Lenart, Tomasz Trzepieciński, Andrzej Krzysiak, Wojciech Łabuński

**Affiliations:** 1 Department of Manufacturing and Production Engineering, Faculty of Mechanical Engineering and Aeronautics, Rzeszow University of Technology, Al. Powst. Warszawy 8, 35-959 Rzeszów, Poland; akubit@prz.edu.pl; 2 Walter, Pustyny, ul. Księża 83, 38-422 Krościenko Wyżne, Poland; lukas.lenart@gmail.com; 3 Department of Aerospace Engineering, Faculty of Mechanical Engineering and Aeronautics, Rzeszow University of Technology, Al. Powst. Warszawy 8, 35-959 Rzeszów, Poland; a.krzysiak@prz.edu.pl; 4 Department of Applied Mechanics and Robotics, Faculty of Mechanical Engineering and Aeronautics, Rzeszow University of Technology, Al. Powst. Warszawy 8, 35-959 Rzeszów, Poland; w.labunski@prz.edu.pl

**Keywords:** flash welding, sandblasting, static strength, surface engineering, tool steel

## Abstract

The aim of the research presented in this article is analysis of the effect of the surface treatment method on the static and fatigue strength of flash butt welded bandsaw blades. A 1-mm-thick 75Cr1 cold-work tool steel sheet used for bandsaw blades was used as the test material. Fractographic studies of the fatigue fractures and fractures formed in static tests were also carried out. The static strength tests showed sandblasting the weld surface had no significant effect on the load capacity of the joint. However, the sandblasted specimens showed a higher repeatability of the load capacity (lower standard deviation). In the case of both analyzed sample variants of specimens, sandblasted and non-sandblasted, the number of cycles at which the sample was damaged decreases with the percentage increase of the stress amplitude. When loading the samples with a stress amplitude value in the range between 400 and 690 MPa, sandblasting of the weld surface increased the average value of destructive cycles by about 10–86% (depending on the stress amplitude) compared to non-sandblasted joints. The sandblasting process introduces compressive stresses in the surface layer of the welds, therefore the variable tensile load acting on the sample requires a greater number of cycles before the fatigue cracks initiate and propagate. In the case of all specimens, a ductile fracture was observed. It was also found that, regardless of the variable stress amplitude, sandblasting has a positive effect on reducing the standard deviation of fatigue test results.

## 1. Introduction

Welding technology is widely used in various industries because it is much cheaper than other joining technologies. Welded joints have much wider applications; it is still one of the basic methods of joining materials in the automotive [1] and machine industries [2]. In the engineering industry, spot welded joints are of great use because machine covers are often large and made of thin sheets [3]. In the production of shields from thin sheets, the use of resistance welding is much cheaper than, for example, the use of fusion welding [4]. It is a similar situation in the automotive industry, where the use of welded joints is more economic, and often used for complex shape components.

Bandsaw machines are the basic equipment of machining plants, allowing for the precise cutting of a wide range of materials, including wood [5], stone [6], non-ferrous metals, stainless steels, cast iron, heat-resistant alloys and structural steels [7]. Taking into account the functionality of cutting machines, it can be stated that the following models are available on the market: manual, gravity, semi-automatic and automatic controlled devices. The bandsaw blade is usually guided on the basis of ball bearings or hydraulically pressed sintered carbide guides. An inverter provides the optimal bandsaw blade speed in relation to the processed material for a wide range of values, most often 10–120 m/min.

So far in world industry, the known and used method of permanently joining bandsaw blades is the process of flash butt welding (FBW). FBW represents an attractive welding process due to its high productivity and wide applicability [8]. The main advantage of the using FBW for bandsaw blades is the possibility of making a permanent joint of very high quality whose mechanical properties are not less than those of the base material [9]. Apart from the proper welding of the bandsaw blade, its service life is largely determined by the proper conduction of the running-in and cooling processes [10]. Bandsaw blade steel must have a good balance between strength, toughness and material elasticity in the weld joint [9]. Workpieces sensitive to water can be lubricated with an oil spray which is applied directly to the bandsaw blades. During the bandsaw blade’s running-in period, reducing the optimal working speed of the belt to 70% and the feed rate to 50% should be taken into account. Bandsaw blades are produced in a thickness range of about 0.65–1.3 mm and a width of 3–80 mm, with various tooth profiles, as well as having a constant or variable pitch, various blade types (uniform, bimetal and with sintered carbide blades) and various protective coatings [11].

Analysis of the FBW of bandsaw blades is the subject of a number of research works. Kalincová [12] analyzed the influence of different annealing temperatures on the structural and mechanical properties of C75 steel welded bandsaw blades. The results of microstructure evaluation confirmed the need for annealing after welding bandsaw blades. Gochev [13] investigated the processes of tempering bandsaw blades after welding according to both weld methods—Metal Inert Gas (MIG) and Metal Active Gas (MAG). It was found that the homogeneity of a joint and the diffusion between the basic metal (the bandsaw blade) and the secondary material (the welding wire) is improved using the MIG and MAG methods. Bodea et al. [9] flash butt welded 51CrV4 steel strips used for manufacturing bandsaw blades and concluded that minor changes in the welding parameters or in the post-welding treatment can cause significant changes in the bandsaw blade’s durability and performance. Ichiyama and Kodama [14] studied the effects of welding conditions and base metal chemical compositions on the flash butt weld defects of high strength steels. They concluded that although flash welding is an efficient welding method, it has limited applications because of the difficulties involved in ensuring the required weld quality. Krishnaraj et al. [15] studied the quality of flash butt welded joints in mild steel. The results indicated that increases in the preflashing energy and preheating energy improve the weld quality significantly. There are many methods of hardening to increase the durability of the bandsaw blades: electro-contact hardening [16], rigging the tool teeth with carbide plates and tempering the teeth in a high-frequency current field [17] and electro spark processing [18].

In addition to flash butt welding, endless bandsaw blades can also be joined by brazing, gas welding, as well as MIG and TIG welding [13]. In the MIG and TIG methods, a very high temperature is produced, which weakens the microstructure of the material, thus reducing the fatigue life of the joint. The TIG welding of the endless saw blades is economically efficient in the case of small series production, as well as for repairing broken blades during their exploitation [19]. The ends of the bandsaw blades are joined by means of an overlay brazing process, which requires high level brazing skills, but still produces a weak joint on account of the foreign material introduced into it. The electric resistance butt welding is the modern method where the joint strength is 25% higher than that of the base metal. This process is also characterized by high speeds and is automatic, thus eliminating human error and producing a perfectly strong joint [20]. The resistance butt welding machine does not require additional flux or solder. After setting the bandsaw blades and achieving a correct clamping by means of the special, quick-acting clamps with which the machine is equipped, the welding process takes place automatically with the assumed welding parameters. Compared to the brazing and MIG processes, the flash butt welding technology has the advantages of being very low-priced and of lacking any foreign material introduced into the weld joint. Moreover, the preparation of the weld surface is not required. Metals with different melting temperatures can be welded using this flash welding process. Flash welding is mostly used for welding steel but can also be used for aluminum alloys, magnesium alloys, stainless steels, low-alloy steels, tool steels, heat resisting alloys, Ni-based alloys, Cu-based alloys and Ti-based alloys. Compared to other joining methods, flash butt welding is suitable for mass production. A solid phase, forge weld is made, and any molten metal and contaminants formed at the interface during heating are squeezed out into the upset. Thus, solidification cracking and porosity are not normally an issue [21].

Bandsaw blades work under specific load and stress conditions [22]. The blade of the bandsaw machine is subjected to many dynamic, cyclically repetitive forces resulting from the resistance of the cut material. Additionally, it usually works in very variable temperature ranges because of the strong heating of the blade material due to friction [23]. These extremely severe conditions for the bandsaw blade material can cause the blades to break in the places where they are joined. Therefore, the durability of the flash butt weld directly affects the efficiency of production processes which use bandsaws [24].

The fatigue strength of the bandsaw blades is the basic parameter that determines the efficiency and failure-free unfolding of the cutting process. Currently, scientific investigations are focused on ensuring the adequate strength of the joint by optimizing high-energy methods such as MIG and TIG. Meanwhile, the flash butt welding process is still the most economic method of joining bandsaw blades in mass production. The introduction of a fast, non-energy-consuming and low-priced method that would increase the strength of welds and would prevent the formation of by-products is desirable in the machine industry. The methods of mechanically increasing the strength of the material in cold forming conditions fit perfectly into the above-mentioned quality indicators. Due to the obvious need to increase the fatigue life of welded joints using low-cost surface treatment methods in the joint area, the heat-affected zone and the base material, it is important to understand the mechanisms influencing the increase in fatigue strength. As a consequence of the surface treatment methods leading to changes in the value of the residual stress, which in turn affects the initiation and development of fatigue cracks, it is important to conduct a thorough analysis resulting in a specific determination of the optimal values and characteristics of the applied stresses in the subsurface area. This, in turn, should be precisely correlated with the parameters of the surface treatment used. Considering the above statements, it is justified to undertake research works aimed at methods that improve the durability and fatigue strength of joints, and at the same time accurately characterize the parameters leading to such improvement.

The purpose of this work is to analyze the effect of sandblasting the flash butt weld surface on the static and fatigue strength properties of the joints made of 75Cr1 steel, which is commonly used for bandsaw blades. The thickness of the strips does not exceed 1 mm [16], therefore FBW is currently the dominant technology for producing bandsaw blades. Moreover, which is equally important in the production of bandsaws, FBW is cheap, and in addition, the joints are characterized by adequate strength and are very easy to make. This article considers the possibility of increasing the strength of 1-mm-thick metal sheet joints by sandblasting, which, according to the best of the authors’ knowledge, has not been studied so far. The sandblasting process, by elastic-plastic deformation, creates compressive stress in the surface layer of the flash butt weld. It also leads to strain hardening in the outer layer of the weld material. The samples were subjected to fatigue tests with different levels of stress amplitude.

## 2. Materials and Methods

### 2.1. Material

The test material used was 1-mm-thick 75Cr1 cold-work tool steel. This steel is commonly used in the wood industry for the production of bandsaws, circular saws and equipment which requires high abrasion resistance. The chemical composition of the 75Cr1 steel, according to the ISO 4957:2018 standard [25], is shown in Table 1.

### 2.2. Flash Butt Welded Specimens

**Figure 1:**
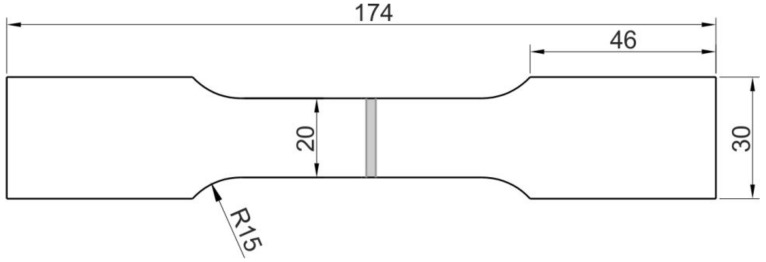
Dimensions (in mm) of the flash butt welded specimen for strength testing.

The samples for the static and fatigue testing of the welded joints, in the form of dog-bone specimens with dimensions of 174 mm × 30 mm (Figure 1), were cut using laser processing on a STX 2500 machine (Yamazaki Mazak Corporation, Takeda, Japan). The laser processing parameters were:

- laser power P = 1700 kW,
- frequency f = 500 Hz,
- cutting speed v = 2700 mm/s,
- gas pressure 0.4 bar.

The laser cutting parameters were consistent with the conditions for cutting bandsaws and circular saws made of 75Cr1 sheets.

**Figure 2:**
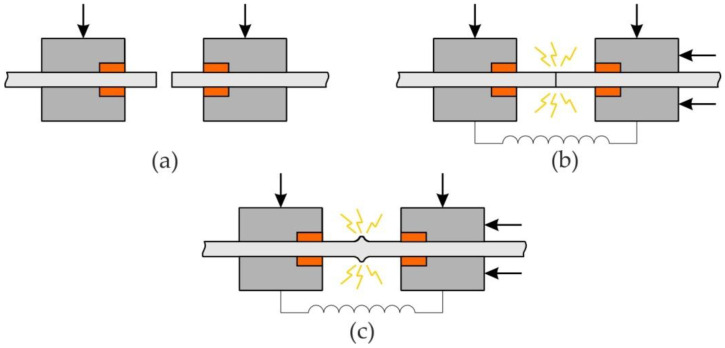
Flash butt welding of a bandsaw blade: (**a**) fixing the sheets in the device, (**b**) switching on the current flow with pressure, (**c**) ending the welding process.

The FBW machine for bandsaw blades used in the experiments was a Viscat VC 4 (Fulgor s.r.l., Torino, Italy). The edges of the bands to be joined must be clean in the area of the butt. After carefully setting the edges of the bands to be joined in the welding device (Figure 2a), with the force ensuring a stable position of the bands during the welding process, the Cu-Cr alloy electrode is pressed with appropriate force against the upper surface of the bandsaw blade. After the welding pressure is exerted, a current of appropriate intensity is passed through the electrodes and the joint (Figure 2b). Under the influence of current flow, the resistive heating transforms the joint area into a highly plasticized state, and the pressure force upsets the welding area (Figure 2c), ensuring a high-quality joint with mechanical properties not lower than the base material.

The parameters of the FBW process (power absorbed 4.5 kW, welding time 5 s) corresponded to the parameters used in the production of bandsaw blades by the manufacturer Walter (Krościenko Wyżne, Poland). After welding, the faces of the welds were grinded.

### 2.3. Fatigue Strength Testing

Fatigue strength tests of flash butt welded specimens were carried out on an HT-9711 Dynamic Testing Machine (Hung Ta Instrument Co., Taichung City, Taiwan). The fatigue tests were carried out at room temperature with a limited number of cycles equal to 2 × 10^6^ and a frequency of 50 Hz. The coefficient of the stress cycle of *R* = 0.1 was used which corresponds to a tension-tension cycle in which *σ_min_* = 0.1*σ_max_* [26]. In order to compare the fatigue strength, sandblasted and non-sandblasted samples were tested. All variants of the specimens were tested for five levels of dynamic loading. At every level, the tests were repeated four times. The lowest level of dynamic load was the value at which the specimen did not fail after being loaded by 2 × 10^6^ cycles. Five specimens were tested for each level of amplitude.

### 2.4. Sandblasting Procedure

Sandblasting was carried out on a KCW 1000 machine (New-Tech, Dobrzykowice, Poland). Processing parameters: sandblasting pressure p = 2 atm, abrasive—GH50 cast steel shot, nominal fraction d = 0.3 mm, abrasive hardness—approx. 60–68 HRC. The sandblasting treatment was aimed not only at cleaning the surface, but also (in the places where the weld was made) introducing compressive stresses in the weld subsurface to strengthen the material.

### 2.5. Fractographic Analysis

Fracture morphologies of selected specimens were analyzed using an S-3400 scanning electron microscope (SEM) from Phenom ProX (Nanoscience Instruments, Phoenix, AZ, USA).

## 3. Results and Discussion

### 3.1. Static Strength

**Figure 3:**
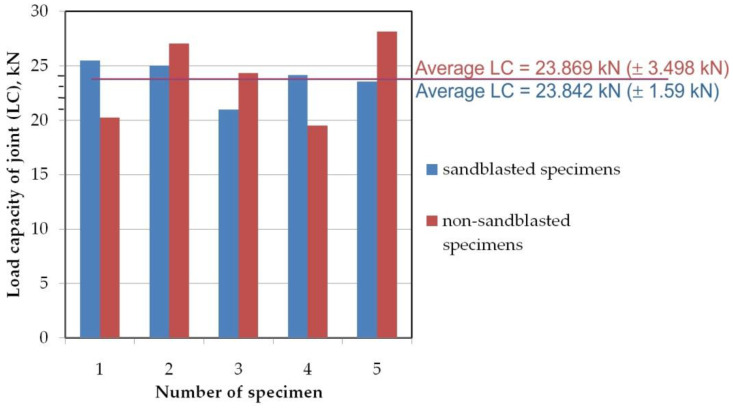
Load capacity of flash butt welded joints.

**Figure 4:**
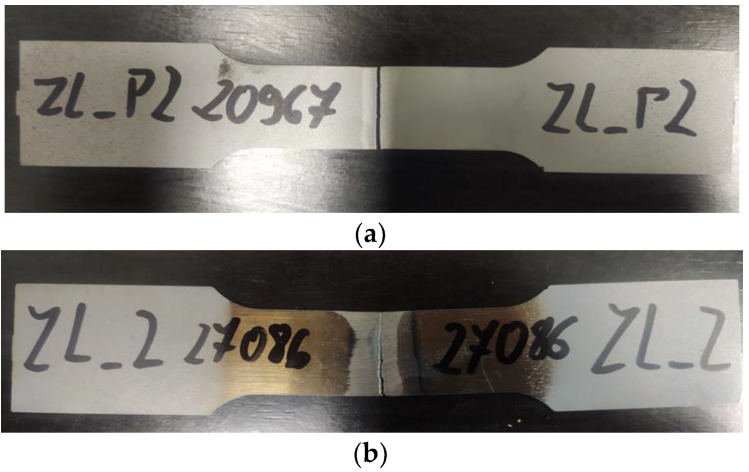
View of the fracture mode of (**a**) sandblasted and (**b**) non-sandblasted butt welds.

In the static tests five specimens were tested for each of the two variants of specimens: sandblasted and non-sandblasted. Based on the results for all repetitions the average load capacity has been determined. The average load capacity (LC) of non-sandblasted flash butt welded joints was approximately 23.8 kN (Figure 3). The static strength tests of the sandblasted sheets did not show any significant influence of this type of treatment on the joint load capacity. However, sandblasted specimens exhibit greater repeatability for load capacity. The standard deviation of the load capacity of these joints was two times smaller than for non-sandblasted samples. All samples were damaged in the weld zone (Figure 4). The results of the statistical analysis of the static tests results for the non-sandblasted and sandblasted specimens are shown in Table 2.

### 3.2. Fatigue Strength

**Figure 5:**
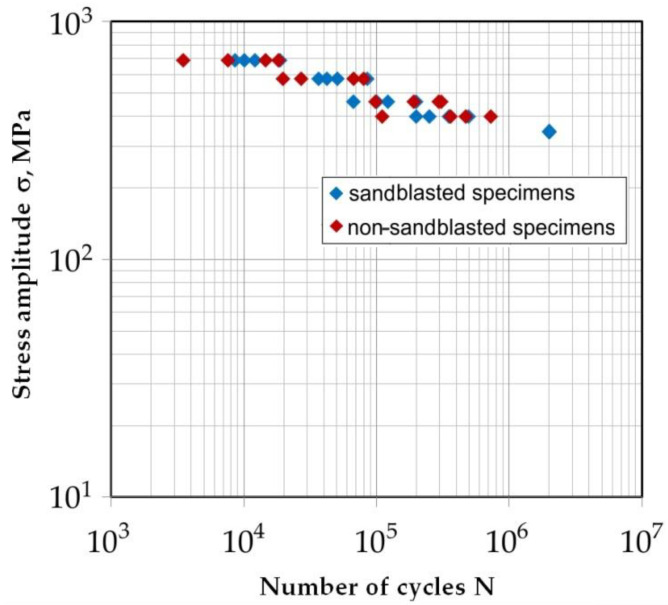
Comparison of the fatigue strength of sandblasted and non-sandblasted specimens.

The common method of characterizing the fatigue performance of welded joints under cyclic loading it to use the Wöhler’s curve. Comparison of the fatigue strength of sandblasted and non-sandblasted specimens is shown in Figure 5. Specimens were tested at five levels of stress amplitude σ: 690 MPa, 575 MPa, 460 MPa, 400 MPa and 345 MPa which correspond to the 100%, 83%, 67%, 58% and 50% of the assumed maximum stress amplitude. At every level, the tests were repeated four times. No sample loaded with an amplitude of 373 MPa was damaged after 2 × 10^6^ cycles. The results of the quantitative analysis of the parameters of the fatigue tests for non-sandblasted and sandblasted specimens are shown in Table 3 and Table 4, respectively. The coefficient of variation *W*_s_ has been determined according to the formula:

(1) $$W_{s}=\frac{s}{log\bar{N}}\times 100\%$$

where *s* is the standard deviation and $\bar{N}$ is the average value of the destructive cycles.

In the case of both analyzed sample variants, the number of cycles at which the sample is damaged decreases with the percentage increase of the set stress. Thus, as the amplitude of the stress increases, the strength of the welded joint decreases, and thus the service life of the bandsaw blades is reduced. Under the load of the samples with the highest amplitude value (σ = 690 MPa), sandblasting of the sample surface increased the average value of the destructive cycles by 12.7%. A similar increase in the mean value of the destructive cycles was observed for sandblasted samples loaded with a stress amplitude of σ = 575 MPa. The load of the sandblasted samples with an amplitude of 460 MPa increased the average value of destructive cycles by about 82% compared to the samples not subjected to sandblasting. The largest difference in the average value of destructive cycles was observed for samples loaded with a stress amplitude of σ = 375 MPa. Sandblasting increased this number by over 86% compared to non-sandblasted samples loaded with the same stress amplitude. This phenomenon can be explained by the differences in the mechanisms of low- and high-cycle fatigue. In the low-cycle fatigue range, plastic deformations resulting from the load hysteresis occur with each cycle, thus plastic deformations accumulate. On the other hand, the sandblasting process introduces compressive stresses in the subsurface layer of the weld, therefore, due to these additional stresses, the tensile variable load acting on the sample requires a greater number of cycles before the fatigue cracks initiate and propagate.

The range of high-cycle fatigue, on the other hand, is characterized by cyclically repeating elastic deformations, in this case, the compressive stresses introduced by sandblasting in the subsurface zone are not of significant importance. At the same time, high-cycle fatigue phenomena are more sensitive to any surface defects. The increase in the repeatability of the fatigue life for the high-cycle fatigue load range can be explained primarily by the uniformity of the surface properties of the samples in terms of geometry and surface roughness. Samples fabricated under industrial conditions and not subjected to sandblasting are characterized by the presence of various types of surface defects with little repeatability, such as micro-grooves and scratches of various shapes and directions, which constitute surface stress concentrators significantly affecting the high-cycle fatigue mechanism. The variety of surface defects may influence the phenomenon of fatigue crack initiation; hence, the non-sandblasted samples exhibit large dispersion of average value of destructive cycles The aforementioned defects are minimized or removed in the sandblasting process, which contributes to increasing the repeatability of the fatigue test results for this sample variant.

Based on the research, it was shown that, regardless of the variable load level, sandblasting has a positive effect on reducing the scatter of the test results, and the samples are subject to fatigue failure in a more reproducible manner. This is due to the standardization of the surface topography and the state of the stresses in the weld subsurface.

### 3.3. Morphology of Fractured Samples upon Quasi-Static Fatigue Loading

**Figure 6:**
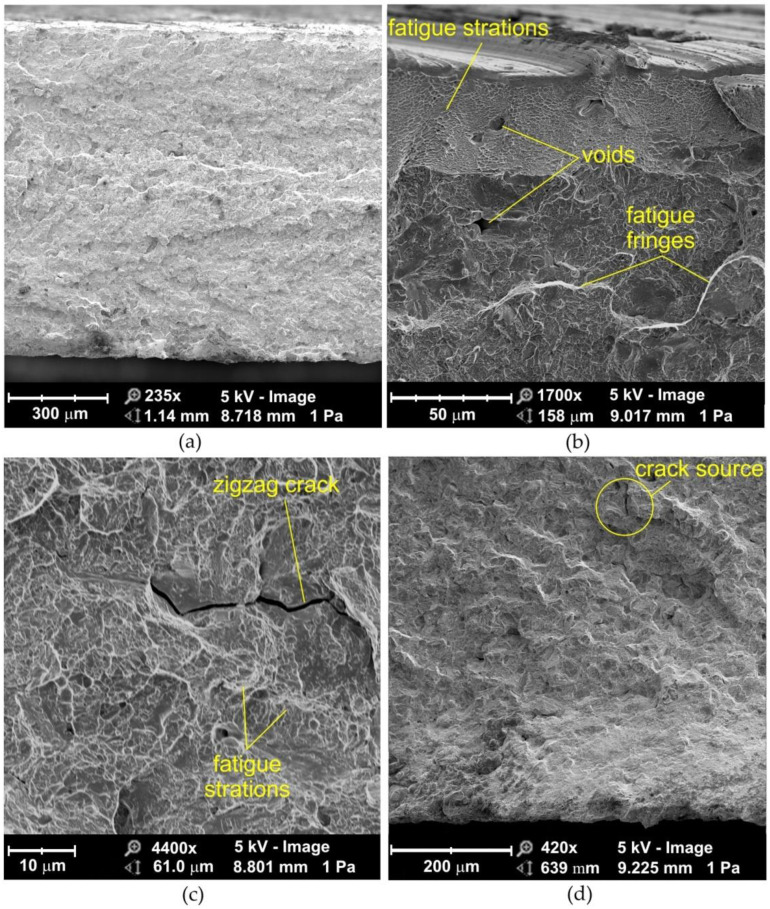
SEM micrographs of the fracture surface of non-sandblasted flash butt welded joints tested at a stress amplitude of 690 MPa: (**a**) cross-section of the fractured surface, (**b**) view of the near-edge layer of the weld, (**c**,**d**) magnification of the middle area of the weld.

**Figure 7:**
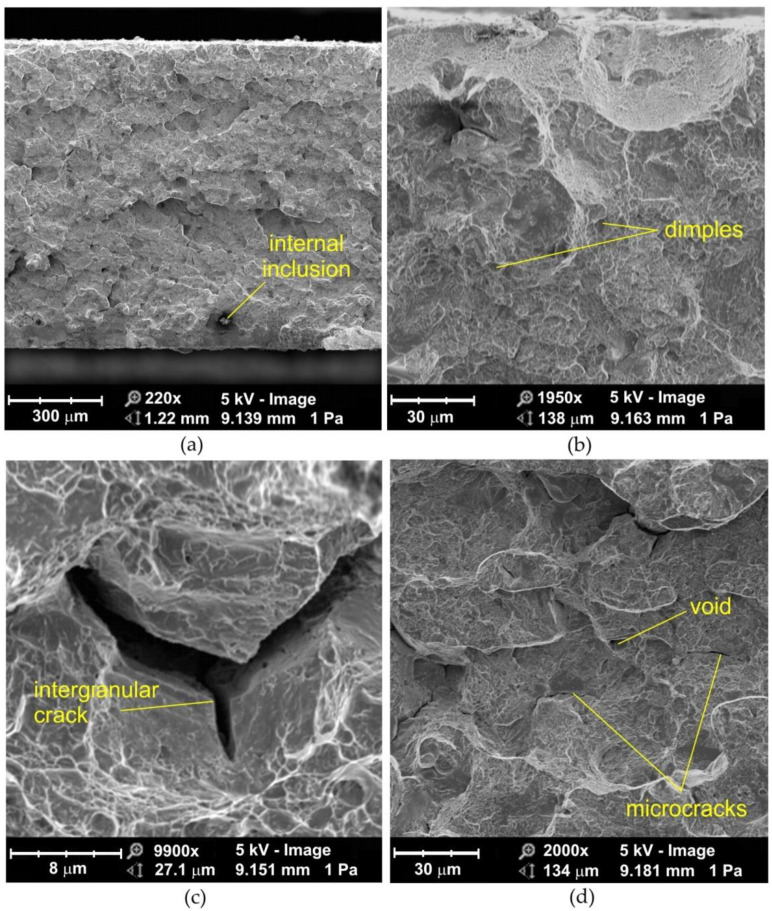
SEM micrographs of the fracture surface of sandblasted flash butt welded joints tested at a stress amplitude of 690 MPa: (**a**) cross-section of the fractured surface, (**b**) view of the near-edge layer of the weld, (**c**,**d**) magnification of the middle area of the weld.

Observation of the fatigue fractures of samples tested with a stress amplitude of σ = 690 MPa showed the destruction is as a result of ductile fracture mode (Figure 6 and Figure 7). During ductile fracture, the formation and joining of cracks takes place due to the plastic flow of the material. Ductile cracking occurs by nucleation and void growth and usually begins with particles of a different phase [27]. Voids are created during solidification stage. Cooling of the nugget takes place immediately after the end of the heating cycle. The solidification front begins from the periphery and moves toward center [28]. Dendrites growing in electrode direction will experience a higher cooling rate and would grow faster than the dendrites growing in the direction of interface/bulk. As a result, the dendrites growing in electrode direction obstruct the interdendritic feeding during the final stages of solidification owing to dendrite coherency [29]. The coherency causes an acute shortage in liquid feeding to the nugget center. This shortage along with metal contraction is believed to cause large pores in the nugget known as shrinkage voids [30,31]. The void growth occurs by the emission of shear dislocation loops from the void surface, which evolve by cross slip to prismatic loops leading to an increase in the void dimensions [32,33]. Turnage et al. [27] found that tensile results indicate that the microstructural damage accumulation due to the change in number density of voids is much faster in the fusion zone than in the parent material, where the change in void growth is the more dominant. The effect of void growth from inclusions is much more prominent in the parent material than in the fusion zone, which shows less ductile behavior (nucleation dominant).

The diameter of the voids is a characteristic structural dimension for the cracking mechanism through the growth and merging of voids [34,35]. This mechanism is determined by the law of the evolution of voids in the stress field in the presence of plastic strains [36,37]. Voids are formed around heterogeneity in the microstructure of the material, that is, carbides and non-metallic inclusions. At room temperature, the voids increase as a result of the development of plastic deformation.

Differences in the strains of the hard particles and the matrix cause the generation of dislocations in the matrix during deformation. If brittle particles of a different phase are present in the tough matrix, such particles are unable to accommodate large plastic deformations of the matrix. Therefore, even when the plastic deformations of the matrix are not very large, the stress caused by external forces reaches a value sufficient for particle fracture [38]. In the near-edge layer of sandblasted joints (Figure 7b), a clear flattening of the traces caused by grinding is visible (Figure 7a). Virtually the entire fracture surface is composed of dimples characteristic of ductile fracture. Dimples in the near-edge layer are spread over large flat surfaces, while in the middle area of the weld, the fracture surfaces show a random character (Figure 6d and Figure 7d) with a few void-initiated fractures (Figure 6c and Figure 7c).

### 3.4. Morphology of Fractured Samples upon Low-Cycle Fatigue Loading

**Figure 8:**
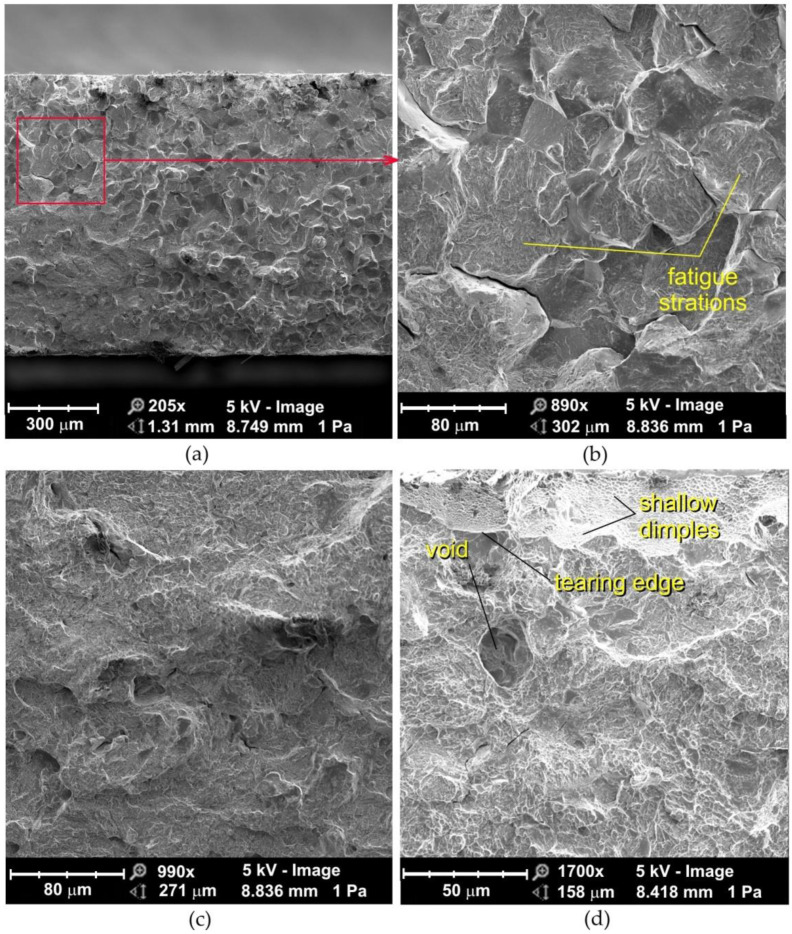
SEM micrographs of the fatigue fracture of non-sandblasted flash butt welded joints tested at a stress amplitude of 575 MPa: (**a**) cross-section of the fatigue fracture, (**b**) magnification of the subsurface area, and (**c**,**d**) view of the near-edge layer of the weld.

**Figure 9:**
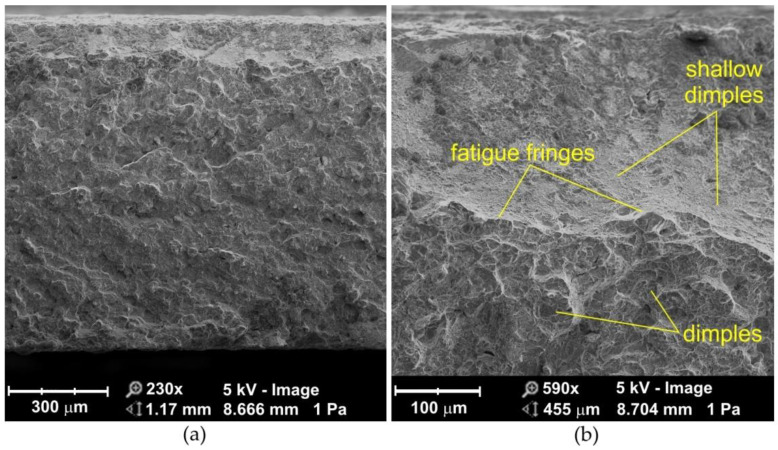
SEM micrographs of the fatigue fracture of sandblasted flash butt welded joints tested at a stress amplitude of 575 MPa: (**a**) cross-section of the fatigue fracture, (**b**) view of the near-edge layer of the weld.

The morphologies of the fatigue fractures formed in low-cycle fatigue conditions (Figure 8 and Figure 9) are characterized by a non-uniform random structure along the entire width of the fatigue fracture (Figure 8a). At high magnification, voids appear, which were formed during the process of joining thermally plasticized materials. This superficial cracking is possibly caused by hydrogen which is deposited in the weld [4]. Hydrogen, diffused from the welding zone to the heat affected zone, accumulates in the discontinuities under the grains. At the same time, hydrogen is pressurized as a gas that generates high internal stresses. Such a type of fracture is common in flash butt welded high strength structural steels [39].

The sandblasted samples in the vicinity of the fracture edge contain a network of dimples smaller in size than in the center of the flash butt weld (Figure 9a,b). Compressive stresses occur in the near-edge zone subjected to sandblasting. These stresses add to those resulting from plastic deformation and the resulting stress sign is reoriented in the subsurface layer. During further deformation of the weld material, local necks are formed between the micro-voids, and when breaking cause, the joining of the voids formed on the particles [38,40]. The processes associated with ductile fracture are usually related to particles of a different phase and the strength of the particle-matrix interface. This type of fracture, characterized by the presence of micro-voids, is due to the coalescence of microcracks that form the nuclei of microcracks in discontinuous areas and are associated with dislocations, second-phase particles, grain boundaries and inclusions [41]. As the deformation increases, the microcracks increase and eventually form a continuous fracture.

### 3.5. Morphology of Fractured Samples upon High-Cycle Fatigue Loading

**Figure 10:**
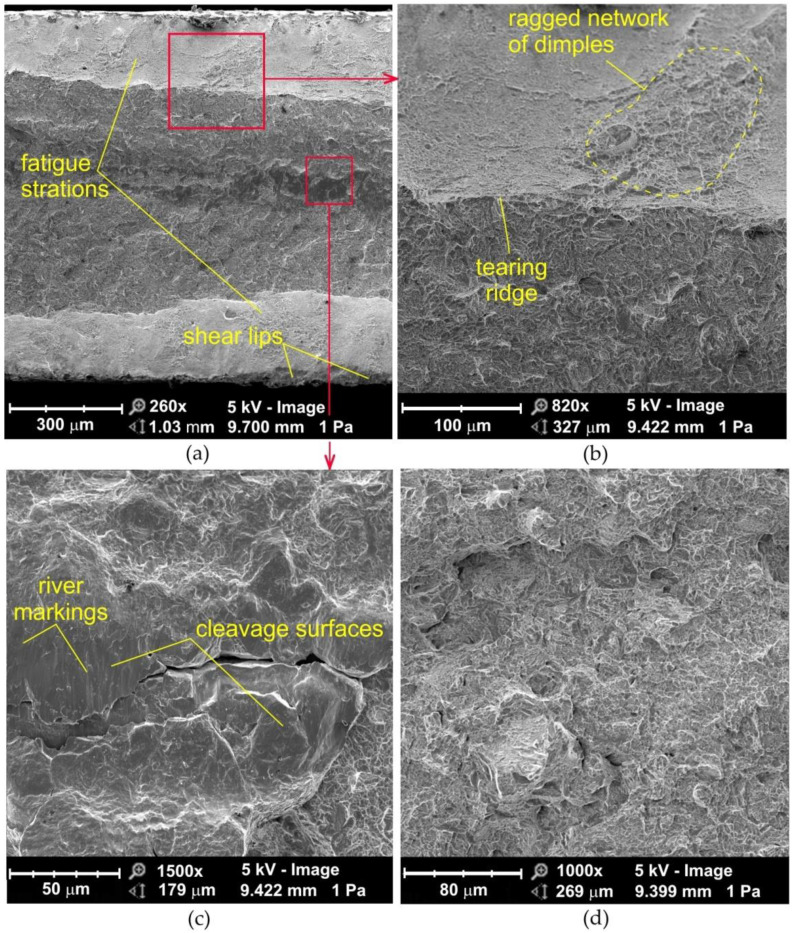
SEM micrographs of the fatigue fracture of non-sandblasted flash butt welded joints tested at a stress amplitude of 460 MPa: (**a**) cross-section of the fatigue fracture, (**b**) magnification of the subsurface area, (**c**,**d**) magnification of the middle area of the weld.

The fatigue fractures of non-sandblasted samples tested under high-cycle fatigue conditions can be divided into two clear zones (Figure 10a): the area adjacent to the edge of the flash butt weld and the middle zone with a mixed fracture mode which corresponds to two ductile fracture mechanisms. In the central part of the fatigue fracture, the crack propagates according to the void growth and merging mechanism (Figure 10d). In general, the center of the weld is devoid of inclusions that could be a source of crack initiation. The studies of Siddiqui et al. [42] showed that the inclusions are pushed out of the area to be welded towards the outer surface of the weld during the upsetting process. At the edges of the weld, on inclined planes, the crack develops according to the shear mechanism (Figure 10a). These inclined planes are called shear lips.

In the sample that is stretched, before formation of the neck begins, micro-voids may form in the entire volume of the sample [43]. During ductile fracture, the tensile strength of the material is less than the stress required to propagate the fracture, therefore the specimen first deforms uniformly, then a neck is formed. Once the neck has begun to form, further deformation and the merging of the voids is confined to this zone. A crack is then formed in the central part of the sample due to the voids merging, and the final separation of the material is achieved by the fracture in the outer areas of the weld.

The ultimate tensile strength of flash butt welded joints is usually 10 to 20% lower than that of the base material, due to the presence of impurities in the weld and significant grain growth in the relatively wide welding zone [44]. Furthermore, deeper zones in the weld cool down more slowly and microstructural transitions take place with some delay. In this way, the material located below the subsurface of weld is stretched, which causes additional compression of the material located directly below this zone. The tensile and compressive stresses add up, causing a specific state of resulting stresses, which depends on the type of material and the welding parameters.

**Figure 11:**
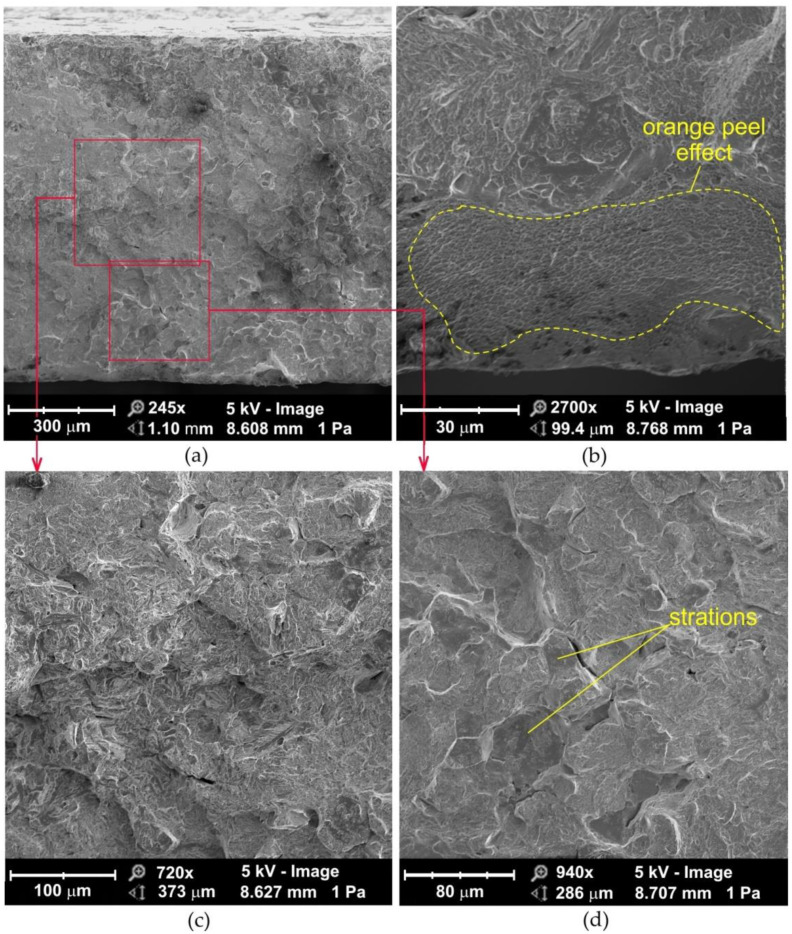
SEM micrographs of the fatigue fracture of sandblasted flash butt welded joints tested at a stress amplitude of 460 MPa: (**a**) cross-section of the fatigue fracture, (**b**) view of the near-edge layer of the weld, (**c**) magnification of the middle area of the weld, (**d**) magnification of the subsurface area of the weld.

The fatigue fracture of a sandblasted specimen subjected to high-cycle fatigue consists of evenly distributed voids (Figure 11) with decreasing size towards the weld edge (Figure 11b) and clear slip planes (Figure 11d). If the majority of the particles on which the voids are formed are located at the grain boundaries, cracking occurs along the grain boundaries (Figure 10c and Figure 11d) and is called intercrystalline.

The fracture mechanism along the slip planes during the development of the ductile fracture is characteristic for high plasticization of the material in front of the crack [45]. Several changes in the fracture mechanism from ductile to brittle can occur during crack growth. With this type of crack development, a brittle fracture is realized only in limited areas of the material, surrounded by the dominant ductile fracture mechanism (Figure 10c).

## 4. Conclusions

The conducted experimental studies on the fatigue strength of flash butt welded joints in 75Cr1 cold-work tool steel allow the following conclusions to be drawn:

- Static strength tests showed no significant effect of sandblasting of the flash butt weld surface on the load capacity of the joint.
- The sandblasted samples were characterized by a greater repeatability of the static load capacity determined by the value of the standard deviation.
- In the case of both analyzed sample variants (sandblasted and non-sandblasted), the number of cycles at which the sample is damaged decreases with the percentage increase of the stress amplitude.
- Depending on the stress amplitude value, sandblasting of the weld surface increased the average value of destructive cycles by about 10–86% (depending on the stress amplitude) compared to samples not subjected to sandblasting.
- Regardless of the variable amplitude level, sandblasting has a positive effect on reducing the distribution of the test results, with the samples subjected to fatigue failure in a more reproducible manner.
- The surfaces of the fatigue fractures formed in low-cycle fatigue conditions are characterized by the ductile fracture mode with an uneven grain structure along the entire width of the fatigue fracture.
- The fatigue fractures of non-sandblasted samples tested under high-cycle fatigue conditions can be divided into those in an area adjacent to the flash butt weld edge and those in a middle zone with a mixed fracture mode. In the central part of the weld, the crack propagates according to the void growth and merging mechanism, and at the near-edge layer of the weld the crack develops according to the shear mechanism.

The tests conducted to determine the suitability of the sandblasting process in increasing the fatigue strength of bandsaw blades have in fact demonstrated, for both analyzed sample variants, that the number of cycles after which the sample was damaged is higher in the case of sandblasted specimens. Future studies should investigate the effect of a wide range of changes in sandblasting parameters (nominal fraction of cast steel shots and sandblasting pressure) on the work hardening of the weld subsurface. The next task will be to determine the effect of sandblasting on the surface roughness of the joint, which under the conditions of high-cycle fatigue may be the source of microcracks initiation. The mechanical properties of the weld material and its microstructure may affect the susceptibility of the joint to work hardening. An interesting research direction may be to determine the fatigue strength of sandblasted joints made by other methods, such as brazing, gas welding, MIG and TIG welding. More extensive research is also planned to analyze the mechanism of strengthening the joints by surface treatment. For this purpose, measurements of residual stresses will be carried out, making possible the determination of the surface treatment parameters for the residual stress distribution, which will then be correlated with the influence of these properties on the fatigue life of the joints. The research plans also assume the determination of the influence of other surface treatment methods on fatigue properties, such as pneumatic ball peening and brushing. Finally, the analysis of the influence of residual stress distribution in subsurface layers on the propagation rate of fatigue cracks will be carried out. The directions of these studies are extremely important, as the relatively low-cost surface treatment can lead to a significant increase in the fatigue life of structural joints. The research results presented in this paper are an introduction to a broad analysis of the phenomena occurring during the fatigue process of flash butt welded joints.

## Figures and Tables

**Table 1:** Chemical composition of the 75Cr1 steel (%wt.).

C	Si	Mn	P	S	Cr	Fe
0.70–0.80	0.25–0.50	0.60–0.80	max. 0.03	max. 0.03	0.30–0.40	remainder

**Table 2:** Statistical analysis of the static tests results.

Parameter	Non-Sandblasted Specimens	Sandblasted Speimens
LC of joint, kN	25.5	20.247
	25.047	27.056
	20.967	24.324
	24.148	19.513
	23.547	28.174
Average value of LC, kN	23.842	23.869
Standard deviation s, kN	1.59	3.498
Coefficient of variation *W_s_*, %	6.669	14.655
Value t_a_ for confidence level *p* = 95%	3.182	3.182
t_a_ × s	5.059	11.131

**Table 3:** Results of the statistical analysis of the fatigue tests for non-sandblasted specimens.

Parameter	Values for Individual Specimens				
Stress Amplitude σ, MPa	690	575	460	400	345
Number of destructive cycles *N* × 10^3^	7.586	26.970	121.903	110.263	2000
	14.570	67.508	67.031	473.251	2000
	3.483	80.451	100.492	363.04	2000
	18.246	19.635	198.564	732.53	2000
Logarithmic number of destructive cycles log*N*	3.880	4.431	5.086	5.042	6.301
	4.163	4.829	4.826	5.675	6.301
	3.542	4.905	5.002	4.559	6.301
	4.261	4.293	5.298	4.865	6.301
Average value of destructive cycles $\bar{N}$	10,971	48,641	121,997	173,268	-
Standard deviation *s*	0.27984	0.2588	0.1696	0.40755	-
Coefficient of variation *W_s_*, %	7.063	5.608	3.356	8.093	-
Value *t_a_* for confidence level *p* = 95%	3.182	3.182	3.182	3.182	-
*t_a_* × *s*	0.89	0.823	0.539	1.297	-
log*N*_up_	4.852	5.438	5.592	6.332	-
*N_up_* × 10^3^ cycles	71.139	274.294	391.516	256.204	-
log*N*_low_	3.071	3.791	4.513	3.738	-
*N_low_* × 10^3^ cycles	1.178	6.182	32.614	5.479	-
Fatigue strength *Z_g_* at 2 × 10^6^ cycles	345				

**Table 4:** Results of the statistical analysis of the fatigue tests for sandblasted specimens.

Parameter	Values for Individual Specimens				
Stress Amplitude σ, MPa	690	575	460	400	345
Number of destructive cycles *N* × 10^3^	10.067	42.285	190.749	249.901	2000
	18.723	50.760	98.032	494.720	2000
	12.104	85.458	307.645	351.386	2000
	8.547	36.574	294.482	198.640	2000
Logarithmic number of destructive cycles log*N*	4.003	4.626	5.280	5.397	6.301
	4.272	4.705	4.991	5.694	6.301
	4.083	4.931	5.488	5.545	6.301
	3.932	4.563	5.469	5.298	6.301
Average value of destructive cycles $\bar{N}$	12,360	53,769	222,727	323,661	-
Standard deviation *s*	0.12717	0.13940	0.19960	0.15006	-
Coefficient of variation *W_s_*, %	3.123	2.962	3.761	2.736	-
Value t_a_ for confidence level *p* = 95%	3.182	3.182	3.182	3.182	-
*t_a_* × *s*	0.405	0.443	0.635	0.477	-
log*N*_up_	4.477	5.150	5.942	5.961	-
*N_up_* × 10^3^ cycles	30.004	141.331	875.749	915.155	-
log*N*_low_	3.667	4.263	4.672	5.006	-
*N_low_* × 10^3^ cycles	4.654	18.326	46.999	101.506	-
Fatigue strength *Z_g_* at 2 × 10^6^ cycles	345				

## Data Availability

The data presented in this study are available on request from the corresponding author.

## References

[1] Chvertko Y., Shevchenko M., Pirumov A. (2013). Monitoring of the process of Flash-Butt Welding. Soldag. Insp..

[2] Kim D.C., So W.J., Kang M.J. (2009). Effect of flash butt welding parameters on weld quality of mooring chain. Arch. Mater. Sci. Eng..

[3] Balaev E.Y., Litvinov A.E. (2018). Analysis of modern technologies for improving performance characteristics of cutting band saws. Adv. Eng. Res..

[4] Baracaldo R.R., Santos M.C., Echeverria M.A.A. (2018). Effect of flash butt welding parameters on mechanical properties of wheel rims. Sci. Tech..

[5] Borz S.A., Oghnoum M., Marcu M.V., Lorincz A., Proto A.R. (2021). Performance of Small-Scale Sawmilling Operations: A Case Study on Time Consumption, Productivity and Main Ergonomics for a Manually Driven Bandsaw. Forests.

[6] Ahsan A., Kenney K., Kröger J., Böhm S. (2020). Hydrostatic Bandsaw Blade Guides for Natural Stone-Cutting Applications. J. Manuf. Mater. Process..

[7] Wu S.H., Huang M.S., Jhou C.E., Wei C.C. (2018). Study on the Cutting Efficiency of High-Speed Band Saw Blade by Taylor Tool Life and Fractal Equations. Matec Web Conf..

[8] Wardoyo T.T.B., Izman S., Kurniawan D. (2013). Effect of Butt Joint on Mechanical Properties of Welded Low Carbon Steel. Adv. Mater. Res..

[9] Bodea M., Sechel N., Popa F. (2015). Investigation of the flash butt welding of 51CrV4 steels for saw blades. Adv. Mater. Res..

[10] Orlowski K.A., Dobrzynski M., Gajowiec G., Lackowski M., Ochrymiuk T. (2020). A Critical Reanalysis of Uncontrollable Washboarding Phenomenon in Metal Band Sawing. Materials.

[11] Ni J., Lang J., Wu C. (2017). Effect of surface texture on the transverse vibration for sawing. Int. J. Adv. Manuf. Technol..

[12] Kalincová D. (2012). Analysis of welded joint of band-saw blade—Influence of annealing process on joint microstructure and mechanical properties. Manuf. Technol..

[13] Gochev Z. Tempering of band saw blades after electric arc welding with smelt electrode. Proceedings of the Trieskové a Beztrieskové Obrábanie Dreva Conference.

[14] Ichiyama Y., Kodama S. (2007). Flash-butt welding of high strength steels. Nippon. Steel Tech. Rep..

[15] Krishnaraj N., Rao K.P., Ramachandran E.G. The Quality of Flash Welded Joints in Mild Steel: A Study on the Effects of Welding Parameters. https://citeseerx.ist.psu.edu/viewdoc/download?doi=10.1.1.1050.2245&rep=rep1&type=pdf.

[16] Marinin E., Sergeev D., Marinina N. (2017). The capability of pulsed laser radiation for cutting band saws hardening. Matec Web Conf..

[17] Gochev Z. (2018). Wood Cutting and Cutting Tools.

[18] Mulin Y.I., Kazannikov O.V., Vlasenko V.D. (2011). Hardening of Band Saws with Electrospark Processing.

[19] Mitelea I., Ochian D.D., Burca M., Utu I.D. TIG welding opportunities of bimetallic endless saw blades. Proceedings of the Metal 2011 Conference.

[20] Bandsaw Blade Butt Welder. https://www.cruxweld.com/resistance-welding-equipments/butt-welder/bandsaw-blade-butt-welder/bandsaw-blade-butt-welding-machine/.

[21] What Are the Advantages of Flash (Butt) Welding?. https://www.twi-global.com/technical-knowledge/faqs/faq-what-are-the-advantages-of-flash-butt-welding.

[22] Rubin M.B., Tufekci E. (2006). Contact stress on a rotating elastic band saw blade using the theory of a cosserat surface. J. Mech. Mater. Struct..

[23] Marinov B. (2020). Energy losses in big band saw machines—Analysis and optimization. Arch. Mech. Eng..

[24] Chai H., So C., Yuan P.F. (2021). Manufacturing double-curved glulam with robotic band saw cutting technique. Automat. Constr..

[25] (2018). ISO 4957:2018-Tool Steels.

[26] Kubit A., Drabczyk M., Trzepiecinski T., Bochnowski W., Kaščák Ľ., Slota J. (2020). Fatigue Life Assessment of Refill Friction Stir Spot Welded Alclad 7075-T6 Aluminium Alloy Joints. Metals.

[27] Turnage S.A., Darling K.A., Rajagopalan M., Whittington W.R., Tschopp M.A., Peralta P., Solanki K.N. Quantifying Structure-Property Relationships during Resistance Spot Welding of an Aluminum 6061-T6 Joint. https://arxiv.org/ftp/arxiv/papers/1605/1605.04251.pdf.

[28] Vijayan V., Huda N., Murugan S.P., Chanyoung J., Seung Man N., Namhyun K., Park Y.D. (2017). The weldability study of carbon nanotube based 2nd generation primer coated steel for automotive applications. J. Mech. Sci. Technol..

[29] Vijayan V., Murugan S.P., Son S.G., Park Y.D. (2019). Shrinkage void formation in resistance spot welds: Its effect on advanced high-strength-steel weld strength and failure modes. J. Mater. Eng. Perform..

[30] Pouranvari M., Marashi S.P.H. (2013). Critical review of automotive steels spot welding: Process, structure and properties. Sci. Technol. Weld. Join..

[31] Wan X., Wang Y., Fang C. (2014). Welding defects occurrence and their effects on weld quality in resistance spot welding of AHSS steel. ISIJ Int..

[32] Chang H.J., Segurado J., Rodríguez de la Fuente O., Pabón B.M., Lorca J. (2013). Molecular dynamics modeling and simulation of void growth in two dimensions. Model. Simul. Mater. Sci..

[33] Lubarda V.A., Schneider M.S., Kalantar D.H., Remington B.A., Meyers M.A. (2004). Void growth by dislocation emission. Acta Mater..

[34] Bandstra J.P., Koss D.A., Geltmacher A., Matic P., Everett R.K. (2004). Modeling void coalescence during ductile fracture of a steel. Mater. Sci. Eng. A.

[35] Benzerga A.A., Leblond J.B. (2010). Ductile Fracture by Void Growth to Coalescence. Adv. Appl. Mech..

[36] Neimitz A., Galkiewicz J., Lipiec S., Dzioba I. (2018). Estimation of the Onset of Crack Growth in Ductile Materials. Materials.

[37] Besson J. (2010). Continuum models of ductile fracture: A review. Int. J. Damage Mech..

[38] Long S.L., Liang Y.L., Lu Y.M., Yang M., Yin C.H. (2018). Study on the formation of micro-voids during ductile fracture. Mater. Res. Express.

[39] Bang H., Bang H., Ro C., Jeong S. (2015). Mechanical Behavior of the Weld Joints of Thick Steel Plates Produced by Various Welding Processes. Strength Mater..

[40] Pradhan P.K., Dash P.R., Robi P.S., Roy S.K. (2012). Micro void coalescence of ductile fracture in mild steel during tensile straining. Frat. Integrita Strutt..

[41] Fydrych D., Łabanowski J., Rogalski G. (2013). Weldability of high strength steels in wet welding conditions. Pol. Maritme Res..

[42] Siddiqui M.I.H., Alshehri H., Orfi J., Ali M.A., Dobrotă D. (2021). Computational Fluid Dynamics (CFD) Simulation of Inclusion Motion under Interfacial Tension in a Flash Welding Process. Metals.

[43] Li X., Yang W., Xu D., Ju K., Chen J. (2021). A new ductile fracture criterion considering both shear and tension mechanisms on void coalescence. Int. J. Damage Mech..

[44] Klimpel A. (1999). Welding and Metal Cutting.

[45] Tekoglu C., Hutchinson J.W. (2015). On localization and void coalescence as a precursor to ductile fracture. Phil. Trans. R. Soc. Lond..

